# Intragastric Application of Aspirin, Clopidogrel, Cilostazol, and BPC 157 in Rats: Platelet Aggregation and Blood Clot

**DOI:** 10.1155/2019/9084643

**Published:** 2019-12-30

**Authors:** Sanja Konosic, Mate Petricevic, Visnja Ivancan, Lucija Konosic, Eleonora Goluza, Branimir Krtalic, Domagoj Drmic, Mirjana Stupnisek, Sven Seiwerth, Predrag Sikiric

**Affiliations:** ^1^University Hospital Centre Zagreb, Zagreb, Croatia; ^2^Department of Pharmacology, School of Medicine, University of Zagreb, Zagreb, Croatia; ^3^Department of Pharmacology, Faculty of Medicine, J.J. Strossmayer University of Osijek, Osijek, Croatia; ^4^Department of Pathology, School of Medicine, University of Zagreb, Zagreb, Croatia

## Abstract

We suggest that the stable gastric pentadecapeptide BPC 157 may rescue thrombocyte function. We focused on the antithrombotic agent aspirin, clopidogrel, and cilostazol application in rats; arachidonic acid, ADP, collagen, and arachidonic acid/PGE1 platelet aggregation (aggregometry) and blood clot viscoelastic properties (thromboelastometry); and the pentadecapeptide BPC 157. Rats received intragastrically for three days once daily treatment with antithrombotic agents—aspirin (10 mg/kg) or clopidogrel (10 mg/kg) or cilostazol (10 mg/kg). Medication (BPC 157 (10 *μ*g/kg) or an equal volume of saline (5 ml/kg)) was given intragastrically, immediately after each antithrombotic agent application. For multiple electrode aggregometry and modified rotational thromboelastometry studies, blood sampling was at 2 h after last application. Adenosine diphosphate (ADP test 6.5 *μ*M), arachidonic acid (ASPI test 0.5 mM), a combination of arachidonic acid and prostaglandin E1 (ASPI test 0.5 mM and PGE1-test 30 nM), and collagen (COL test 3.2 *μ*g/ml) were used as aggregation agonists. Given with aspirin, clopidogrel, or cilostazol in rats, BPC 157 counteracted their inhibitory effects on aggregation activated by arachidonic acid, ADP, collagen, and arachidonic acid/PGE1. Specifically, this includes recovery of the aggregation induced by arachidonic acid (*vs.* aspirin, *vs.* clopidogrel, and *vs.* cilostazol), arachidonic acid/PGE1 (*vs.* cilostazol), ADP (*vs.* clopidogrel), or collagen (*vs.* clopidogrel). Contrarily, there is no effect on the used tests (extrinsic/intrinsic hemostasis system, the fibrin part of the clot) EXTEM, INTEM, and FIBTEM; clotting time; clot formation time; alpha-angle; maximum clot firmness; lysis index after 30 minutes; and maximum lysis. In conclusion, we revealed that BPC 157 largely rescues thrombocyte function.

## 1. Introduction

We focused on the inhibitory effect of the stable gastric pentadecapeptide BPC 157 (used in trials: ulcerative colitis; now, multiple sclerosis) [1–13] on the antithrombotic agents (i.e., aspirin, inhibitor of thromboxane A2 (TXA2) production; clopidogrel, P2Y_12_ subtype of adenosine-diphosphate (ADP) receptor antagonist; and cilostazol, phosphodiesterase type 3 (PDE_3_) inhibitor [14]). The effect on platelet aggregation and viscoelastic properties of the blood clot was investigated using multiple electrode aggregometry and modified rotational thromboelastometry (ROTEM) studies [15–20].

Recently, BPC 157 therapy (for review, see [1–13]) approaches solving of the vascular occlusion disturbances [21–25]. The rapid activation of the bypassing loop occurs in the rats with infrarenal occlusion of the inferior caval vein (and thereby resolved Virchow, venous lesion and thrombosis, caval hypertension, aortal hypotension, and consequent thrombocytopenia), much like in the rats with ischemic/reperfusion colitis, duodenal venous congestion lesions, perforated cecum, bile duct ligation-induced liver cirrhosis, and portal hypertension [21–25]. Previously, BPC 157, as a prototype antiulcer agent with potent cytoprotective capability [1–13], thereby exerting innate endothelium protection, counteracted abdominal anastomosis-induced thrombosis [26] and prolonged bleeding and thrombocytopenia after amputation and/or anticoagulant (heparin, warfarin), aspirin, and NO-agents (L-NAME/L-arginine) [27, 28] and largely interacts with NO-system in various models and species [1–13]. While having no effect on noninjured rats or on coagulation parameters, BPC 157 in heparin-treated rats decreased prolonged activated partial thromboplastin time (APTT) but did not influence heparin activity (anti-Xa test) [27].

Thus, we further studied how BPC 157 may influence platelet aggregation and viscoelastic properties of the blood clot. Therefore, these outcomes were carried out using ex vivo and in vitro studies, using impedance aggregometry and ROTEM studies. Rats received intragastrically for three days once daily treatment with antithrombotic agents—aspirin or clopidogrel or cilostazol. Medication (BPC 157 (regular dose of the 10 *μ*g/kg) or saline (controls)) was given intragastrically, immediately after each antithrombotic agent application. In aggregometry studies, arachidonic acid, ADP, collagen, and arachidonic acid/PGE1 were used as aggregation agonists [15–17]. ROTEM studies include CT (clotting time), CFT (clot formation time), and alpha-angle, to indicate the rate of fibrin formation; MCF (maximum clot firmness) to show the platelet contribution to clot formation; Ly30 (lysis index after 30 minutes) and ML (maximum lysis) to show the percentage of lost clot stability; EXTEM test (a screening test for the (extrinsic) hemostasis system); INTEM test (intrinsic pathway is being tested); and FIBTEM (isolates fibrinogen function) [18, 19].

## 2. Materials and Methods

### 2.1. Animals

Male albino Wistar rats, 200 g b.w., were randomly assigned; 6 rats per each group were used for the experiments, approved by the Local Ethics Committee at School of Medicine (University of Zagreb, Zagreb, Croatia). The medication procedure was performed on rats, which had food and water ad libitum before the procedure and until the end of the experiment, and was assessed by the observer unaware about the treatment.

### 2.2. Drugs and Protocol

Pentadecapeptide Gly-Glu-Pro-Pro-Pro-Gly-Lys-Pro-Ala-Asp-Asp-Ala-Gly-Leu-Val, M.W. 1419, named BPC 157, a part of the sequence of human gastric juice protein, coded BPC, freely soluble in water at pH 7.0 and in saline, was prepared (Diagen, Slovenia) as described previously [1–13]. L-NAME and L-arginine were commercially purchased (Sigma, USA).

Aspirin (Andol, Pliva, Croatia), clopidogrel (Zyllt, Krka Ltd., Slovenia), and cilostazol (PLETAL, Otsuka Pharmaceutical Ltd., UK) were used.

Rats received intragastrically for three days once daily treatment with antithrombotic agents—aspirin (10 mg/kg) or clopidogrel (10 mg/kg) or cilostazol (10 mg/kg). Medication (BPC 157 (10 *μ*g/kg) or an equal volume of saline (5 ml/kg)) was given intragastrically, immediately after each antithrombotic agent application. The rats were then sacrificed at 2 h after the last application.

### 2.3. Blood Sampling

In deeply anaesthetized rats (with ketamine (20 mg/kg, Ketanest, Parke Davis GmbH, Germany) and diazepam (10 mg/kg, Normabel, Belupo, Croatia)), a median sternotomy was performed. By direct puncture to the right atrium using a 20G needle, blood was collected into 2.6 ml S-Monovette tubes (Sarsted Ltd., Germany) (final hirudin concentration 25 *μ*g/ml) for aggregometric measurements, and 1.8 ml into 3.8% citrate Vacuette tubes (Greiner Bio-One Ltd., Austria) for thromboelastometric measurements.

### 2.4. Measurements

Platelet aggregation was determined in whole blood by multiple electrode aggregometry (MEA) on Multiplate® Analyzer (Tem International GmbH, Germany). Technical details have already been described in previous literature [15–17]. To put it briefly, MEA is based on the principle that activated platelets stick on the test cell sensor wires and then enhance the electrical resistance between them, which is continuously recorded and expressed as 3 parameters: aggregation (AGG) (highest increase in impedance between the electrodes measured in aggregation units (AU)), area under the curve (AUC) (determined by the height of the aggregation curve and the slope measured in U = AU/min (1 U = 10 AU/min)), and velocity (VEL) (maximum slope of aggregation measured in AU/minute). Measurements were executed according to the manufacturer's instructions, using equipment and kits provided by Dynabyte, Munich, Germany. Four test cells were loaded with 300 *μ*l of normal saline and 300 *μ*l of whole blood, followed by three-minute incubation at 37°C. After the incubation, 20 *μ*l of the agonist was added to each respective cell: via ADP receptors by ADP (ADP test 6.5 *μ*M) [17]; by arachidonic acid, the substrate of cyclooxygenase (COX), which subsequently forms the potent platelet activator TXA2 (ASPI test 0.5 mM) [17]; by a combination of arachidonic acid and prostaglandin E1 (ASPI test 0.5 mM and PGE1 test 30 nM), where PGE1 does not affect arachidonic acid-induced platelet aggregation per se but potentiates the inhibitory effects of cilostazol on platelet aggregation in *in vitro* studies [20]; and by collagen via the collagen receptor, which leads to a release of endogenous arachidonic acid and TXA2 (COL test 3.2 *μ*g/ml) [17]. After six minutes of measurement, AUC, AGG, and VEL were recorded.

Viscoelastic properties of the blood were assessed using modified rotational thromboelastometry (TEM) on ROTEM® delta analyzer (Tem International GmbH, Germany). A detailed description of the ROTEM technology has been published previously [18, 19]. In short, TEM measures elasticity and strength of the developing clot in whole blood via a pin suspended in a cup. Changes in the movement of the pin are converted by digital data processing to create graphical and numerical output. Typical parameters obtained are the time from the beginning of measurement until the clot starts to form (CT); the time needed for the clot to reach an amplitude of 20 mm (CFT); alpha-angle, angle of tangent at 2 mm amplitude; the maximum amplitude of the curve during 60 minutes of measurement (MCF); clot lysis at 30 minutes (Ly30); and maximum lysis (ML), which describes the percentage of the maximum lost clot firmness relative to MCF. Following standard analyzer set-up and reagents provided by Tem International GmbH, Germany, 300 *μ*l of citrated whole blood was firstly recalcified with 20 *μ*l of CaCl_2_ 0.2 mol/l (STARTEM). Coagulation was then initiated by adding 20 *μ*l of activator through EXTEM, INTEM, or FIBTEM. After 60 minutes CT, CFT, alpha-angle, MCF, Ly30, and ML were recorded.

### 2.5. Statistical Methods

The normality of the distribution was tested using the Kolmogorov-Smirnov test. The differences between parameters were analyzed using the Kruskal-Wallis test and post hoc analysis using the Mann-Whitney *U* test with Bonferroni correction. All *P* values less than 0.05 were considered significant. In data analysis, StatsDirect statistical software (http://www.statsdirect.com; England: StatsDirect Ltd. 2013) 3.0.171 version was employed.

## 3. Results

### 3.1. Aggregometry Studies

**Figure 1:**
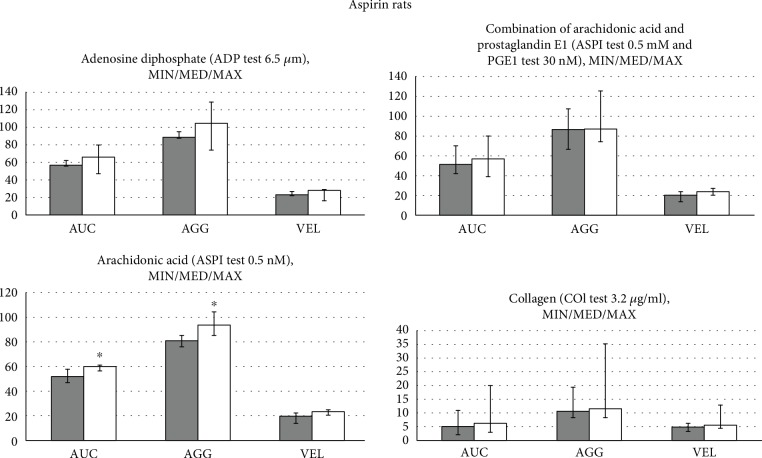
Rats which underwent antithrombotic agent aspirin (10 mg/kg intragastrically, once daily for three days) received immediately thereafter BPC 157 (10 *μ*g/kg intragastrically, once daily for three days) (white bars) or an equal volume of saline (5 ml/kg, intragastrically, once daily for three days) (gray bars); they were sacrificed at 2 h after the last application. Platelet aggregation was determined in whole blood by multiple electrode aggregometry (MEA) on Multiplate® Analyzer (aggregation (AGG)) (highest increase in impedance between the electrodes measured in aggregation units (AU)), area under the curve (AUC) (determined by the height of the aggregation curve and the slope measured in U = AU/min (1 U = 10 AU/min)), and velocity (VEL) (maximum slope of aggregation measured in AU/minute). After the incubation, 20 *μ*l of the agonist was added to each respective cell: adenosine diphosphate (ADP test 6.5 *μ*M), arachidonic acid (ASPI test 0.5 mM), a combination of arachidonic acid and prostaglandin E1 (ASPI test 0.5 mM and PGE1 test 30 nM), and collagen (COL test 3.2 *μ*g/ml). After six minutes of measurement, AUC, AGG, and VEL were recorded. ^∗^*P* < 0.05, *vs.* control, at least.

**Figure 2:**
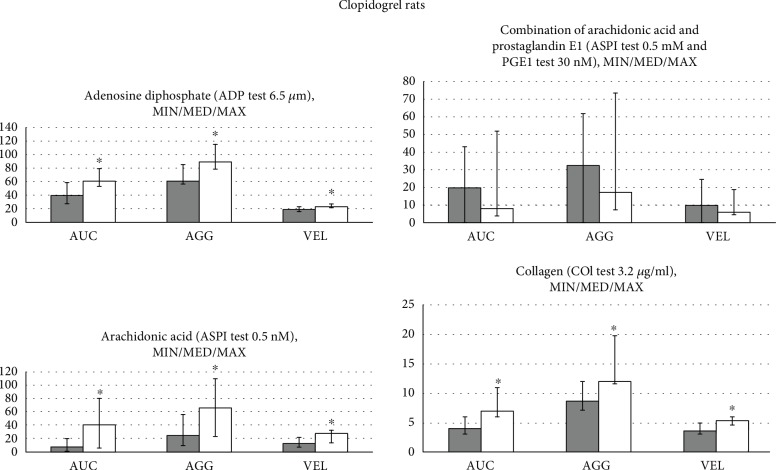
Rats which underwent antithrombotic agent clopidogrel (10 mg/kg intragastrically, once daily for three days) received immediately thereafter BPC 157 (10 *μ*g/kg intragastrically, once daily for three days) (white bars) or an equal volume of saline (5 ml/kg, intragastrically, once daily for three days) (gray bars); they were sacrificed at 2 h after the last application. Platelet aggregation was determined in whole blood by multiple electrode aggregometry (MEA) on Multiplate® Analyzer (aggregation (AGG)) (highest increase in impedance between the electrodes measured in aggregation units (AU)), area under the curve (AUC) (determined by the height of the aggregation curve and the slope measured in U = AU/min (1 U = 10 AU/min)), and velocity (VEL) (maximum slope of aggregation measured in AU/minute). After the incubation, 20 *μ*l of the agonist was added to each respective cell: adenosine diphosphate (ADP test 6.5 *μ*M), arachidonic acid (ASPI test 0.5 mM), a combination of arachidonic acid and prostaglandin E1 (ASPI test 0.5 mM and PGE1 test 30 nM), and collagen (COL test 3.2 *μ*g/ml). After six minutes of measurement, AUC, AGG, and VEL were recorded. ^∗^*P* < 0.05, *vs.* control, at least.

**Figure 3:**
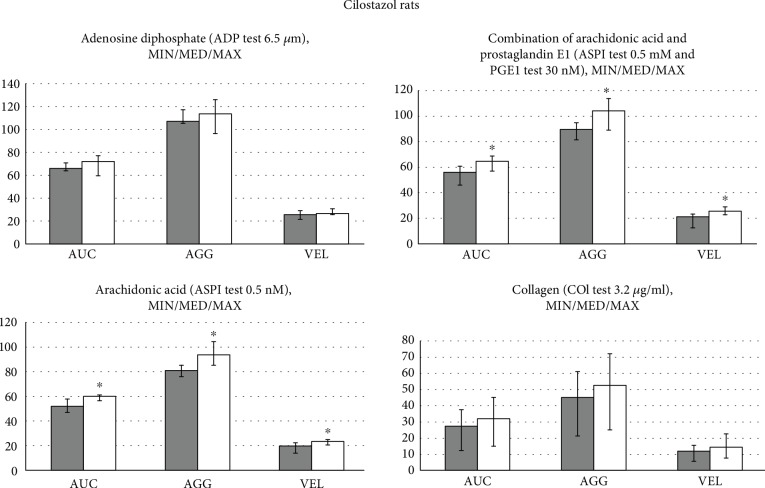
Rats which underwent antithrombotic agent cilostazol (10 mg/kg intragastrically, once daily for three days) received immediately thereafter BPC 157 (10 *μ*g/kg intragastrically, once daily for three days) (white bars) or an equal volume of saline (5 ml/kg, intragastrically, once daily for three days) (gray bars); they were sacrificed at 2 h after the last application. Platelet aggregation was determined in whole blood by multiple electrode aggregometry (MEA) on Multiplate® Analyzer (aggregation (AGG)) (highest increase in impedance between the electrodes measured in aggregation units (AU)), area under the curve (AUC) (determined by the height of the aggregation curve and the slope measured in U = AU/min (1 U = 10 AU/min)), and velocity (VEL) (maximum slope of aggregation measured in AU/minute). After the incubation, 20 *μ*l of the agonist was added to each respective cell: adenosine diphosphate (ADP test 6.5 *μ*M), arachidonic acid (ASPI test 0.5 mM), a combination of arachidonic acid and prostaglandin E1 (ASPI test 0.5 mM and PGE1 test 30 nM), and collagen (COL test 3.2 *μ*g/ml). After six minutes of measurement, AUC, AGG, and VEL were recorded. ^∗^*P* < 0.05, *vs.* control, at least.

BPC 157, given immediately after antithrombotic agents in rats (aspirin, inhibitor of TXA2 synthesis; clopidogrel, ADP receptor antagonist; and cilostazol, selective PDE_3_ inhibitor), counteracted their inhibitory effects on aggregation activated by arachidonic acid, ADP, collagen, and arachidonic acid/PGE1, which were used as aggregation agonists (Figures 1–3).

In general, while aggregation responses to arachidonic acid, ADP, collagen, and arachidonic acid/PGE1 were observed in all animals, some particularities consistently appear. Maximal AUC, AGG, and VEL values obtained with collagen were lower in the aspirin rats (Figure 1) and in the clopidogrel rats (Figure 2) than in the cilostazol rats (Figure 3). Maximal AUC, AGG, and VEL values obtained with arachidonic acid or arachidonic acid and prostaglandin E1 were lower in the clopidogrel rats (Figure 2) and in the aspirin rats (Figure 1) than in the cilostazol rats (Figure 3). The platelet agonist ADP-induced maximal AUC, AGG, and VEL values were comparable in the aspirin rats (Figure 1), clopidogrel rats (Figure 2), and cilostazol rats (Figure 3), and ADP is therefore considered as a most common agonist.

#### 3.1.1. Aspirin Rats

It is likely indicative that BPC 157 reversed the aspirin effect on the arachidonic acid-induced platelet aggregation (maximal AUC and AGG), since arachidonic acid-induced platelet aggregation is typically inhibited by aspirin. However, the rescuing effect on the maximal AUC, AGG, and VEL induced by ADP, arachidonic acid and prostaglandin E1, and collagen did not reach the level of the significance (Figure 1).

#### 3.1.2. Clopidogrel Rats

It is likely indicative that BPC 157 reversed the effect of clopidogrel on the ADP-induced platelet aggregation since ADP-induced platelet aggregation is typically inhibited by clopidogrel. Interestingly, BPC 157 reversed also the effect of clopidogrel on the maximal AUC, AGG, and VEL induced by arachidonic acid or collagen. The rescuing effect on the arachidonic acid and prostaglandin E1 did not reach the level of the significance (Figure 2).

#### 3.1.3. Cilostazol Rats

It is likely indicative that BPC 157 reversed the effect of cilostazol on the arachidonic acid- and arachidonic acid and prostaglandin E1-induced platelet aggregation. Namely, arachidonic acid- and arachidonic acid and prostaglandin E1-induced platelet aggregation is typically inhibited by cilostazol. The rescuing effect on the ADP and collagen did not reach the level of the significance (Figure 3).

### 3.2. Rotational Thromboelastometry

**Figure 4:**
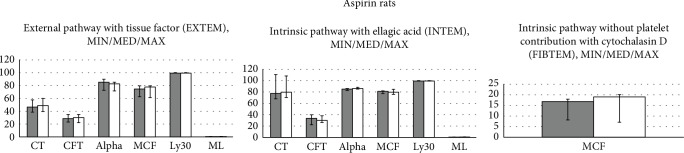
Rats which underwent antithrombotic agent aspirin (10 mg/kg intragastrically, once daily for three days) received immediately thereafter BPC 157 (10 *μ*g/kg intragastrically, once daily for three days) (white bars) or an equal volume of saline (5 ml/kg, intragastrically, once daily for three days) (gray bars); they were sacrificed at 2 h after the last application. Viscoelastic properties of the blood were assessed using modified rotational thromboelastometry (TEM) on ROTEM® delta analyzer (Tem International GmbH, Germany). Typical parameters obtained are clotting time (CT), the time from the beginning of measurement until the clot starts to form; clot formation time (CFT), the time needed for the clot to reach an amplitude of 20 mm; alpha-angle, angle of tangent at 2 mm amplitude; maximum clot firmness (MCF), the maximum amplitude of the curve during 60 minutes of measurement; Ly30, clot lysis at 30 minutes; and maximum lysis (ML) which describes the percentage of the maximum lost clot firmness relative to MCF. We analyzed the external pathway with tissue factor (EXTEM), an intrinsic pathway with ellagic acid (INTEM), or without platelet contribution with cytochalasin D (FIBTEM). After 60 minutes, CT, CFT, alpha-angle, MCF, Ly30, and ML were recorded. *P* > 0.05, *vs.* control.

**Figure 5:**
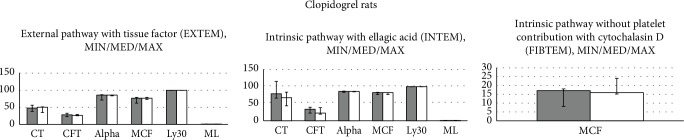
Rats which underwent antithrombotic agent clopidogrel (10 mg/kg intragastrically, once daily for three days) received immediately thereafter BPC 157 (10 *μ*g/kg intragastrically, once daily for three days) (white bars) or an equal volume of saline (5 ml/kg, intragastrically, once daily for three days) (gray bars); they were sacrificed at 2 h after the last application. Viscoelastic properties of the blood were assessed using modified rotational thromboelastometry (TEM) on ROTEM® delta analyzer (Tem International GmbH, Germany). Typical parameters obtained are clotting time (CT), the time from the beginning of measurement until the clot starts to form; clot formation time (CFT), the time needed for the clot to reach an amplitude of 20 mm; alpha-angle, angle of tangent at 2 mm amplitude; maximum clot firmness (MCF), the maximum amplitude of the curve during 60 minutes of measurement; Ly30, clot lysis at 30 minutes; and maximum lysis (ML) which describes the percentage of the maximum lost clot firmness relative to MCF. We analyzed external pathway with tissue factor (EXTEM), an intrinsic pathway with ellagic acid (INTEM); or without platelet contribution with cytochalasin D (FIBTEM). After 60 minutes, CT, CFT, alpha-angle, MCF, Ly30, and ML were recorded. *P* > 0.05, *vs.* control.

**Figure 6:**
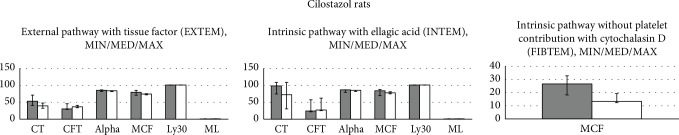
Rats which underwent antithrombotic agent cilostazol (10 mg/kg intragastrically, once daily for three days) received immediately thereafter BPC 157 (10 *μ*g/kg intragastrically, once daily for three days) (white bars) or an equal volume of saline (5 ml/kg, intragastrically, once daily for three days) (gray bars); they were sacrificed at 2 h after the last application. Viscoelastic properties of the blood were assessed using modified rotational thromboelastometry (TEM) on ROTEM® delta analyzer (Tem International GmbH, Germany). Typical parameters obtained are clotting time (CT), the time from the beginning of measurement until the clot starts to form; clot formation time (CFT), the time needed for the clot to reach an amplitude of 20 mm; alpha-angle, angle of tangent at 2 mm amplitude; maximum clot firmness (MCF), the maximum amplitude of the curve during 60 minutes of measurement; Ly30, clot lysis at 30 minutes; and maximum lysis (ML) which describes the percentage of the maximum lost clot firmness relative to MCF. We analyzed external pathway with tissue factor (EXTEM), an intrinsic pathway with ellagic acid (INTEM), or without platelet contribution with cytochalasin D (FIBTEM). After 60 minutes CT, CFT, alpha-angle, MCF, Ly30, and ML were recorded. *P* > 0.05, *vs.* control.

By contrast, neither of the used tests, EXTEM, INTEM, and FIBTEM, found any effect on CT, CFT, alpha-angle, MCF, Ly30, and ML (Figures 4–6).

Thus, these studies demonstrated in the thrombocytes after antithrombotic agents' application, with distinctive targets (TXA2 inhibition-ADP receptor inhibition-selective PDE_3_ inhibition), distinctive failures in the particular functions (as may be seen with distinctive aggregation responses to arachidonic acid, ADP, collagen, and arachidonic acid/PGE1) (Figures 1–3). Having no effect on the coagulation pathways (Figures 4–6), BPC 157 corroborates vice versa with typical antithrombotic agents' targets (TXA2 inhibition-ADP receptor inhibition-selective PDE_3_ inhibition) (Figures 1–3) rescuing the aggregation activated by the arachidonic acid, ADP, collagen, and arachidonic acid/PGE1.

## 4. Discussion

We demonstrated that BPC 157 medication [1–13], given immediately after antithrombotic agents in rats, aspirin, clopidogrel, and cilostazol, counteracted their inhibitory effects on the aggregation activated by the arachidonic acid, ADP, collagen, and arachidonic acid/PGE1 used as aggregation agonists [15–17, 20]. Contrarily, BPC 157 does not affect coagulation pathways; neither of the used tests, EXTEM, INTEM, and FIBTEM, did find any effect on CT, CFT, alpha-angle, MCF, Ly30, and ML. Thus, this suggests a particular pleiotropic effect, likely a direct effect, on thrombocyte function. This may follow since BPC 157 counteracted abdominal aorta anastomosis or inferior caval vein occlusion-induced thrombosis [21, 26], prolonged bleeding and thrombocytopenia after amputation and/or anticoagulant (heparin, warfarin), aspirin, and NO-agents (L-NAME/L-arginine), or prolonged venous occlusion [21, 27, 28]. This may be particularly important considering the effectiveness with intragastric application, as a general follow-up of its cytoprotection background (i.e., antiulcer peptide, native and stable in human gastric juice more than 24 h) [1–13]. On the other hand, AUC is affected by both velocity and maximum aggregation and is considered as the best parameter to reflect overall platelet aggregation [17], and results are dependent on both platelet count and platelet function [17]. Therefore, there are distinctive presentations of the arachidonic acid-, ADP-, arachidonic acid/PGE1-, and collagen-induced aggregation in the aspirin, clopidogrel, and cilostazol rats. Consequently, we suggest typical distinction (TXA2 inhibition-ADP receptor inhibition-selective PDE_3_ inhibition), which results with distinctive platelet dysfunctions, depending on the typical antithrombotic agent use, aspirin, clopidogrel, or cilostazol, and vice versa, on the applied therapy effect.

Namely, considering the BPC 157-aspirin counteracting relation (rescued arachidonic acid aggregation), if BPC 157 is given, thrombocyte function may resist against the effect of aspirin, which otherwise irreversibly blocks the formation of TXA_2_ in platelets [14]. Therefore, in the aspirin rats cured with BPC 157 application, there is the viability of the thromboxane pathway. Much like this, with respect to BPC 157-clopidogrel counteracting relation, with BPC 157, thrombocytes may function despite the effect of clopidogrel, which otherwise specifically and irreversibly inhibits the P2Y_12_ subtype of ADP receptor [14]. Therefore, ADP likely binds to two G protein-coupled receptors P2Y1 and P2Y12 and initiates primary wave platelet aggregation through calcium mobilization [14]. This may allow in the BPC 157-clopidogrel rat collagen-induced platelet aggregation as a complex multistep process that is dependent on the release of ADP and thromboxane from platelets to amplify the response [29]. Similarly, with respect to the BPC 157-cilostazol counteracting relation, BPC 157 rescues thrombocyte function against cilostazol action and thereby against selective inhibition of PDE_3_, an increase in cAMP, as well as against an increase in the active form of protein kinase A (PKA), which is directly related to an inhibition in platelet aggregation [14]. Therefore, we demonstrated in the BPC 157-cilostazol rats that arachidonic acid-induced platelet aggregation appears, otherwise most effectively inhibited by cilostazol ex vivo in previous reports [30, 31]. Moreover, in these BPC 157-cilostazol rats, rescue of the arachidonic acid and PGE1 aggregation also occurs, where PGE1 would otherwise potentiate the inhibitory effects of cilostazol on platelet aggregation in in vitro studies [32]. These beneficial effects on the typical targets of the antithrombotic agents may be present despite the other particular platelet function which may be still disturbed. Illustratively, in the aspirin rats, there is no significant rescue of the maximal AUC, AGG, and VEL induced by ADP, arachidonic acid and prostaglandin E1, and collagen. In clopidogrel rats, no significant rescue of the arachidonic acid and prostaglandin E1 occurs. In cilostazol rats, there is no significant rescue of the ADP and collagen.

In support, while the complete BPC 157 mechanisms remain to be fully determined (several molecular pathways affected, in particular VEGFR2-receptors) [2, 21, 33–38], BPC 157 may consolidate prostaglandin system function [1–13]. This may be also a follow-up of Robert's cytoprotection concept general understanding and applicability (i.e., epithelium/endothelium maintenance *vs.* prostaglandin-system inhibition by NSAIDs) [39] and BPC 157 role as the novel cytoprotection mediator [1–13]. It is therefore also logical to expect that thrombocyte function will be also maintained. BPC 157 largely reversed various NSAID-toxicities, after both COX 1 and COX 2 inhibitors, also gastrointestinal, liver, and brain lesions that appeared after their overdose(s) application(s) [8, 40–43], and has its own particular anti-inflammatory effect (BPC 157 counteracts the increase of the proinflammatory and procachectic cytokines [2]), and may both prevent and reverse adjuvant arthritis in rats [44]. Furthermore, BPC 157/NO-relationship is established in various experimental models and species, providing that it might interfere with the effects of either NOS-blockade or NOS-substrate agent application [1–12], and thereby consolidate NO-system toward better healing effect, thus maintaining platelet function along with the endothelium maintenance. Likely, such balanced thrombocyte function may also contribute to the beneficial effect obtained in the rats with occluded blood vessels [21–25]. Rapid activation of the bypassing loop occurs with BPC 157 therapy in the rats with the infrarenal occlusion of the inferior caval vein (and thereby resolved Virchow, venous lesion and thrombosis, caval hypertension, aortal hypotension, and consequent thrombocytopenia) [21]. It occurs much like in the rats with ischemic/reperfusion colitis, duodenal venous congestion lesions, perforated cecum, bile duct ligation-induced liver cirrhosis, and portal hypertension [22–25].

In conclusion, BPC 157 may exert a particular effect on thrombocyte function, being a promising agent in further application. This should be however seen with known limitations (i.e., the effect of acetylsalicylic acid and clopidogrel does not have any influence on thromboelastometry/thromboelastography, as well [45]).

## Data Availability

The data used to support the findings of manuscript 9084643 titled “Intragastric Application of Aspirin, Clopidogrel, Cilostazol, and BPC 157 in Rats: Platelet Aggregation and Blood Clot” are included within the article.
